# SMSP Mainlobe Jamming Suppression with FDA-MIMO Radar Based on FastICA Algorithm

**DOI:** 10.3390/s23125619

**Published:** 2023-06-15

**Authors:** Pengfei Wan, Guisheng Liao, Jingwei Xu, Xiaolong Fu

**Affiliations:** 1 National Key Laboratory of Radar Signal Processing, Xidian University, Xi’an 710071, China; gsliao@xidian.edu.cn (G.L.); jwxu@xidian.edu.cn (J.X.); 2 Air and Missile Defence College, Air Force Engineering University, Xi’an 710051, China; fuxiaolong_12@163.com

**Keywords:** smeared spectrum jamming, frequency diverse array, multiple-input multiple-output, maximum entropy method, blind source separation

## Abstract

In the electronic warfare environment, the performance of ground-based radar target search is seriously degraded due to the existence of smeared spectrum (SMSP) jamming. SMSP jamming is generated by the self-defense jammer on the platform, playing an important role in electronic warfare, making traditional radars based on linear frequency modulation (LFM) waveforms face great challenges in searching for targets. To solve this problem, an SMSP mainlobe jamming suppression method based on a frequency diverse array (FDA) multiple-input multiple-output (MIMO) radar is proposed. The proposed method first uses the maximum entropy algorithm to estimate the target angle and eliminate the interference signals from the sidelobe. Then, the range-angle dependence of the FDA-MIMO radar signal is utilized, and the blind source separation (BSS) algorithm is used to separate the mainlobe interference signal and the target signal, avoiding the impact of mainlobe interference on target search. The simulation verifies that the target echo signal can be effectively separated, the similarity coefficient can reach more than 90% and the detection probability of the radar is significantly enhanced at a low signal-to-noise ratio.

## 1. Introduction

Linear frequency modulation (LFM) signal is a commonly used waveform for target detection in ground-based radars, which effectively solves the conflict between radar energy and distance resolution [1,2]. However, with the development of electronic warfare technology, enemy aircraft can release self-defense smeared spectrum (SMSP) jamming, which is located in the mainlobe and highly overlaps with the target signal in both time and frequency domains [3,4,5,6]. Through pulse compression processing, multiple dense false targets are obtained, which cover the real target and greatly weaken the detection and tracking performance of ground-based radars. Therefore, how to suppress mainlobe SMSP jamming is an urgent and important problem to be solved [7,8].

It is known that mainlobe jamming is not easy to implement. Nevertheless, it is well known that a self-defensive jammer is a type of equipment that generates mainlobe jamming in a direction exactly consistent with the true target [9,10,11,12,13]. The Electronic Support Measures (ESM) system intercepts the radiated waveform of the radar, and the airborne Electronic Counter Measures (ECM) system generates suppressed or deceived jamming by modulating the frequency, timing and phase of the signal so that the radar cannot detect targets or detect multiple false targets, resulting in a reduction in the search and tracking capability of the radar. In recent years, a new type of mainlobe jamming technology has attracted widespread attention in electronic warfare, namely, mainlobe SMSP jamming. Sparrow and Cikalo (2006) invented mainlobe SMSP jamming to counter pulse compression (PC) radars, which can generate a large number of range false targets (RFTs) in LFM-PC ranging radars. This is a nonstationary time-varying (TV) signal that can be regarded as the sum of multiple chirps [14,15]. SMSP jamming has the same bandwidth as the radar detection waveform, which can obtain the gain of pulse compression to achieve a better effect. Furthermore, SMSP jamming can generate a large number of false targets by controlling the number of sub-pulses in the range dimension. It confuses the radar system about the true target origin and makes the PC radar system ineffective [16].

SMSP jamming is obviously different from the LFM signal. According to this essential feature, the traditional anti-jamming method can use some improved time-frequency analysis methods, such as fractional Fourier transform, to achieve the purpose of identifying interference [17]. In [18], by utilizing the sparsity of fractional Fourier transform (FrFT), a compressed perceptual reconstruction based on fractional domain filtering was proposed to reduce the loss of target echo energy. In [19], the match signal transform (MST) was used to estimate the frequency modulation (FM) rate of the SMSP, and a special matched filter was constructed to calculate the number of sub-pulses of SMSP interference. In [20], a countermeasure against deceptive jamming based on entropy and the parasitic signal characteristics of digital radio frequency memory (DRFM) signals was proposed. However, with the development of DRFM technology, the parasitic signal characteristics are decreasing. Due to the difference in polarization vectors between the jamming signal and the echo signal, a polarizing filter was proposed to suppress jamming [21]. In [22,23], a hybrid polarization method based on interference reconstruction and blind source separation was proposed, but the disadvantage is that it requires the estimation of the slope of the interference signal and the position of the pulse front, which involves a large amount of computation. The combination of classical multiple-input multiple-output (MIMO) technology algorithms with new technologies can effectively improve the anti-interference performance [24,25]. In [26], methods that exploited sequential convex approximation, first-order Taylor expansion and penalty function were proposed, and they could achieve the solutions with a fast convergence rate. Study [27] presented a novel secrecy-energy efficient hybrid beamforming design, which solved the efficiency maximization problem to meet the signal-to-interference-plus-noise ratio (SINR) constraints. The concept of frequency diversity array (FDA) was first proposed by Amtonic P in 2006 and was quickly extended to the field of radar systems [28,29]. By introducing a small frequency increment in the transmit array and combining it with the multiple-input multiple-output (MIMO) technique, FDA-MIMO radars have additional degrees of freedom (DOFs) in the range domain. The FDA-MIMO radar has a two-dimensional dependence on the range and angle, making it attract increased attention in the field of anti-mainlobe jamming. In [30], the principle of FDA-MIMO was first proposed to distinguish between true target and mainlobe deceptive jamming but without considering the time-delay modulation in the deceptive jamming model. In [31], a method for suppressing mainlobe range deceptive jamming was proposed, which mainly utilized pulse diversity to construct orthogonal pulses to distinguish true and false targets at different ranges. The algorithm based on a simulated annealing algorithm was proposed to suppress main-beam range deceptive jamming in [32]. In [33], a mainlobe deceptive interference suppression method based on secondary compensation was proposed. However, all these methods require that the distance and angle information of the target are known.

To solve this problem, this paper proposes a mainlobe interference suppression method based on blind source separation (BSS). The concept of BSS was proposed in the 1980s. At present, BSS based on the maximum signal-to-noise ratio (MSNR) is widely used in speech signal recognition, data communication, image processing, radar array processing and other fields. In [34], the objective function of the blind source separation algorithm based on the maximum signal-to-noise ratio was proposed, and the obtained feature vector was formed into a separation matrix to realize signal separation with low operational complexity. In [35], a method combining radar signal processing and data fusion based on MSNR-BSS was proposed. In [36], an orthogonal frequency division multiplexing–linear frequency modulation–multiple-input multiple-output (OFDM-LFM-MIMO) interference suppression method based on BSS was proposed, which needs further consideration in practical application.

In this paper, an SMSP mainlobe jamming suppression method is proposed with an FDA-MIMO radar system, which effectively improves the target detection performance of the radar. Based on the principle analysis of SMSP interference signals, due to the difference between the interference distance and the signal slope, maximum entropy spectral estimation was used to obtain the target and interference angles, and interference from the sidelobe could be eliminated. Then, based on the obtained azimuth and elevation angles of the target, blind source separation technology was used to solve the signal obtained from this angle, the signal could be distinguished into different channels according to the maximum eigenvalue, and the target distance was obtained through matched filtering. This method can effectively suppress mainlobe SMSP interference without target prior information such as the angle and distance.

The remaining sections are organized as follows. Section 2 presents the fundamentals of an FDA-MIMO radar. The algorithm to suppress SMSP mainlobe jamming within MEM and BSS is explored in Section 3. Simulation and performance analysis results are presented in Section 4. Conclusions are drawn in Section 5.

*Notations*: Boldface is used for vectors *x* (lower case), whose *n*-th entry is [*x*]_n_, and matrices A (upper case). Transpose and conjugate transpose operators are denoted by the symbols (·)^T^ and (·)^H^, respectively. *C^N^*^×1^ and *C^N×M^* are, respectively, the sets of *N*-dimensional vectors of complex numbers and *N* × *M* complex matrices. ⊙ and ⊗ represent the Hadamard product and the Kronecker product, respectively. The letter j represents the imaginary unit (i.e., $j=\sqrt{-1}$). [a, b] indicates a closed interval in real number space. Finally, max{·} and min{·} denote the maximum and minimum values within the feasible set.

## 2. Fundamentals of FDA-MIMO Radar

We consider a uniform planar FDA radar composed of *M × N* antenna elements in horizontal and vertical dimensions. The array element spacing is *d*. Suppose the transmitting and receiving antenna units are omnidirectional radiating, homogeneous and uniform. The signal of the *m*-th transmitter unit can be expressed as [37]

(1) $$s_{m}\left(t\right)=\mathrm{rect}\left(\frac{t}{T_{p}}\right)\varphi_{m}\left(t\right)\exp\left\{j2\pi f_{m}t\right\}$$

where t is the time variable, $\mathrm{rect}\left(\frac{t}{T_{p}}\right)=\left\{\begin{array}{l} 1,0\leq t\leq T_{P}\\ 0,\;\mathrm{else} \end{array}\right.$ is the rectangular window function, where *T_p_* represents a signal pulse, $\varphi_{m}(t)$ is the baseband modulation signal corresponding to the *m*-th transmitter unit, and $f_{m}$ is the transmit frequency corresponding to the *m*-th transmitter unit:

(2) $$f_{m}=f_{0}+(m-1)\Delta f,\begin{array}{c} m=1,2,\ldots ,M \end{array}$$

where $f_{0}$ is the carrier frequency of the reference array element (the first array element), and Δf is the frequency increment.

Assuming that the range and angle parameters of the target are $\left(R,\alpha ,\beta \right)$, where α denotes the azimuth and β denotes the elevation of the target, the target echo transmitted and received by the array element with coordinates $\left(x_{k},y_{k}\right)$ can be expressed as [38]

(3) $$x_{s,k}\left(t-\tau_{k}\right)=\beta_{s0}\mathrm{rect}\left(\frac{t-\tau_{k}}{T_{p}}\right)\varphi_{m}\left(t-\tau_{k}\right)\exp\left\{j2\pi f_{0}\left(t-\tau_{k}\right)\right\}$$

where $\beta_{s0}$ denotes the complex coefficient of the target echo signal containing the full transmitted and received links of the radar. $\tau_{k}$ denotes the difference in the echo time delay between the transmit and receive of the array element at the coordinates $\left(x_{k},y_{k}\right)$. The echo time delay $\tau_{k}$ in a planar array can be expressed as

(4) $$\tau_{k}=\frac{1}{c}\left(x_{k}\cos\alpha \cos\beta +y_{k}\sin\alpha \cos\beta \right)$$

where c denotes the propagation speed of lights.

Since each transmitted array element of the FDA-MIMO radar operates at a different frequency, the phase term introduced by the frequency step cannot be neglected when Equation (3) represents the approximate model under the narrow band assumptions in the far field, and bringing Equation (1) into Equation (3),

(5) $$x_{s,k}\left(t-\tau_{0}\right)\approx \beta_{s0}\mathrm{rect}\left(\frac{t-\tau_{0}}{T_{p}}\right)\exp\left\{j\phi_{m}\left(t-\tau_{0}\right)\right\}\exp\left\{j2\pi \Delta f\left(m-1\right)\left(t-\tau_{k}\right)\right\}\exp\left\{j2\pi f_{0}\left(t-\tau_{k}\right)\right\}$$

where $\tau_{0}=2R/c$ is the reference delay of the target echo. In this way, the target echo signal received by the array element can be approximated as

(6) $$x_{s,k}\left(t-\tau_{0}\right)\approx \sum_{m=1}^{M}\sum_{n=1}^{N}\beta_{s0}\mathrm{rect}\left(\frac{t-\tau_{0}}{T_{p}}\right)\exp\left\{j\phi_{m}\left(t-\tau_{0}\right)\right\}\exp\left\{j2\pi \Delta f\left(m-1\right)\left(t-\tau_{m,n}\right)\right\}\exp\left\{j2\pi f_{0}\left(t-\tau_{m,n}\right)\right\}$$

The echo signal of the target is amplified, matched and filtered, and the distance cell where the target is located can represent the signal as a matrix in the form of

(7) $$s=\beta_{s}\delta \left(t-\tau_{0}\right)a\left(R,\alpha ,\beta \right)⊗b\left(\alpha ,\beta \right)$$

where $\beta_{s}$ denotes the complex coefficients of the target echo after pulse compression, $\delta \left(t-\tau_{0}\right)$ is the sinc function indicating that the target is associated with time delay $\tau_{0}$, and ⊗ denotes the Kronecker product. $a\left(R,\alpha ,\beta \right)$ and $b\left(\alpha ,\beta \right)$ are, respectively, the transmit and receive steering vectors of the target, and they are written as

(8) $$\begin{array}{l} a\left(R,\alpha ,\beta \right)=a_{r}(R)⊙a_{\alpha ,\beta }(\alpha ,\beta )=\\ {\left[1,\exp(-j4\pi \Delta f\frac{R}{c}),\ldots ,\exp(-j4\pi \Delta f\frac{(M-1)R}{c})\right]}^{T}⊙\\ {}[1,\exp(j2\pi \frac{d(\cos\alpha \cos\beta +\sin\alpha \cos\beta )}{\lambda }),\ldots ,\exp(j2\pi \frac{\left(M-1)d(\cos\alpha \cos\beta +\sin\alpha \cos\beta \right)}{\lambda })]=\\ {\left[\begin{array}{l} 1,\exp\left\{-j4\pi \frac{\Delta fR}{c}+j2\pi \frac{d}{\lambda }(\cos\alpha \cos\beta +\sin\alpha \cos\beta )\right\},\cdots ,\\ \ldots \exp\left\{-j4\pi \frac{\Delta fR}{c}\left(M-1\right)+j2\pi \frac{d}{\lambda }\left(M-1\right)(\cos\alpha \cos\beta +\sin\alpha \cos\beta )\right\} \end{array}\right]}^{T} \end{array}$$

(9) $$b\left(\theta \right)={\left[1,\exp\left\{j2\pi \frac{d}{\lambda }(\cos\alpha \cos\beta +\sin\alpha \cos\beta )\right\},\ldots ,\exp\left\{j2\pi \frac{d}{\lambda }\left(M-1\right)(\cos\alpha \cos\beta +\sin\alpha \cos\beta )\right\}\right]}^{T}$$

where ⊙ denotes the Hadamard product, and

(10) $$a_{r}(R)={\left[1,\exp(-j4\pi \Delta f\frac{R}{c}),\ldots ,\exp(-j4\pi \Delta f\frac{(M-1)R}{c})\right]}^{T}$$

and

(11) $$a_{\alpha ,\beta }(\alpha ,\beta )=[1,\exp(j2\pi \frac{d(\cos\alpha \cos\beta +\sin\alpha \cos\beta )}{\lambda }),\ldots ,\exp(j2\pi \frac{\left(M-1)d(\cos\alpha \cos\beta +\sin\alpha \cos\beta \right)}{\lambda })]$$

are, respectively, the range and angle steering vectors.

From Equation (8), it can be seen that the signals received by the FDA-MIMO radar are not only related to the two-dimensional angle in space but also to the target distance. Therefore, the transmit steering vector of the FDA-MIMO radar is angle-range two-dimensionally coupled, and the use of this coupling enables the FDA-MIMO radar to have the ability to distinguish between targets at different distances. This can effectively distinguish between target and jamming signals at different ranges within the same beam, which provides significant practical value against jamming in the mainlobe.

Assuming that the airborne ESM system can detect LFM signals on spatial far-field targets, it can generate multiple interference signals in a fast-time dimension through DRFM storage forwarding and the release of self-defense interference. The power of these interference signals is greater than that of target signals, and they are similar to target signals and exist in the time domain, frequency domain and spatial domain simultaneously. Therefore, the interfering signals received by the $\left(x_{k},y_{k}\right)$ array can be expressed as

(12) $$x_{j,k}\left(t-\tau_{j}\right)=\beta_{j0}\mathrm{rect}\left(\frac{t-\tau_{j}}{T_{p}}\right)\varphi_{m}\left(t-\tau_{j}\right)\exp\left\{j2\pi f_{0}\left(t-\tau_{j}\right)\right\}\exp(f_{j}(t))$$

where $\exp(f_{j}(t))$ denotes the modulation of the modulation function of the SMSP jamming signal (more details in Section 3). $\tau_{j}$ denotes the time delay of the jamming signal, written as

(13) $$\tau_{j}=\frac{1}{c}\left(x_{k}\cos\alpha \cos\beta +y_{k}\sin\alpha \cos\beta \right)+\Delta \tau$$

where Δτ denotes the delay time of the jamming signal relative to the target echo signal. Assuming that the ECM system radiates *P* jamming signals, it can be obtained that the SMSP signals received by the array element can be expressed as

(14) $$j\left(t\right)=\sum_{j=1}^{P}\beta_{j}\delta \left(t-\tau_{j}\right)a\left(\tau_{j},\alpha ,\beta \right)⊗b\left(\alpha ,\beta \right)$$

It can be seen that the jamming signal has the same form as the target echo signal, except that the interference signal has been modulated and the waveform has changed. This requires interference suppression processing during the search process, which depends on the characteristics and interference characteristics of the FDA-MIMO radar, the specific algorithms of which will be analyzed in the next section. Based on a combination of the target echo signal, the interference signal and the noise, the radar-received signal can be expressed as [39]

(15) $$\begin{array}{c} x\left(t\right)=s\left(t\right)+j\left(t\right)+n\left(t\right)\\ \;=\beta_{s}\delta \left(t-\tau_{0}\right)a\left(\tau_{0},\alpha ,\beta \right)⊗b\left(\alpha ,\beta \right)+\sum_{j=1}^{P}\beta_{j}\delta \left(t-\tau_{j}\right)a\left(\tau_{j},\alpha ,\beta \right)⊗b\left(\alpha ,\beta \right)+n\left(t\right) \end{array}$$

where $n\left(t\right)$ is complex Gaussian white noise.

## 3. SMSP Mainlobe Jamming Suppression for FDA-MIMO Radar

The SMSP jamming mainly interferes with the LFM radar. After receiving data, the onboard ESM system processes them through mixing and filtering and stores them in a digital radio frequency memory (DRFM). Then, the data are transferred through a transmission gate and sent to a shift register group in the data buffer area, where the clock frequency of the shift register is N times the clock frequency when the control data are sent to the DRFM. After repeating the data N times, the interference signal is transmitted by the ECM system after analog-to-digital conversion and mixing and filtering. Therefore, the SMSP interference signal is a time-width invariant signal composed of N residuals, and its FM slope is N times that of the radar transmit signal.

### 3.1. Mechanism of SMSP

It is assumed that the LFM of the FDA-MIMO radar transmit is

(16) $$s(t)=\mathrm{rect}(\frac{t-\tau /2}{\tau })\exp\left(2\pi f_{0}t+\frac{1}{2}Kt^{2}\right),\;t\in \left[0,\tau \right]$$

where τ denotes the signal pulse width, K denotes the FM slope, B=Kτ denotes the bandwidth of the signal transmit, and rect(t/τ) denotes a rectangular pulse with τ.

After the airborne ESM system intercepts the radar radiation signal, it is able to generate the first interferer pulse, and the signal can be expressed as [40]

(17) $$s_{J1}(t)=\delta_{J}\mathrm{rect}(\frac{t-\tau /2n-\Delta t-\Delta t_{J}}{\tau /n})\exp\left(j2\pi nf_{0}(t-\Delta t-\Delta t_{J})+j\pi nK{\left(t-\Delta t-\Delta t_{J}\right)}^{2}\right),\;t\in \left[0,\tau \right]$$

where $\delta_{J}$ denotes the amplitude of the jamming signal, and $\Delta t_{J}$ denotes the time delay of the SMSP interference; *n* is the *n*-th jamming pulse; Δt denotes the target echo time delay. The FM slope of the sub-pulse is n times the transmit signal, and the pulse width is 1/n of the original pulse width as shown in Equation (17). This will then be repeated n times in the time domain to obtain the complete SMSP jamming signal model.

(18) $$s_{J}(t)=\sum_{i=1}^{n}s_{J1}(t-i\frac{\tau }{n})=s_{J1}⊗\sum_{i=1}^{n}\delta (t-i\frac{\tau }{n})$$

where ⊗ denotes the convolution operation and δ(t) denotes the impulse function. The instantaneous frequency of the jamming signal can be calculated using the phase derivative of the point at φ(t):

(19) $$f_{J}(t)=\frac{1}{2\pi }\cdot \frac{d\varphi (t)}{dt}=\sum_{i=1}^{n}rect(\frac{t-(2i-1)\tau /2n}{\tau /n})[nKt-(i-1)B]$$

**Figure 1:**
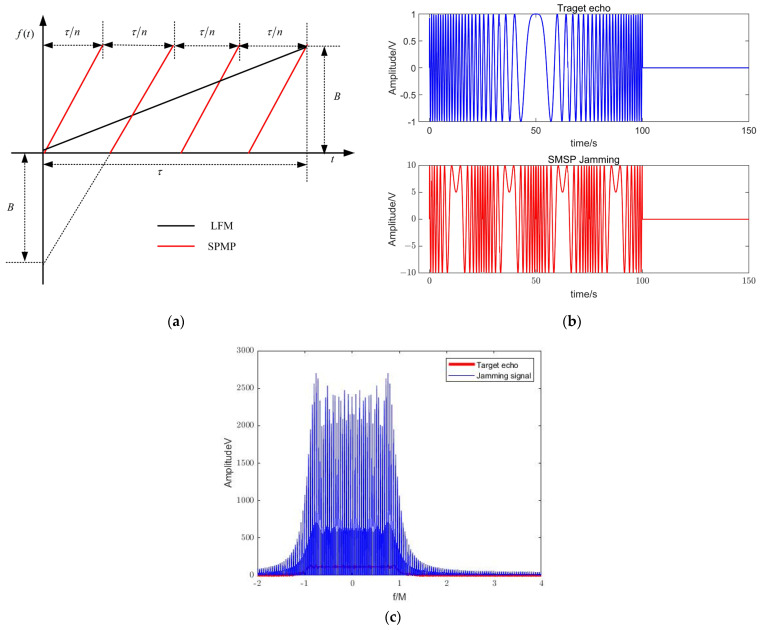
Sketch map of instantaneous frequency of LFM signal and SMSP jamming signal. (**a**) The slopes of LFM and SMSP. (**b**) LFM and SMSP signal in time domain. (**c**) LFM and SMSP signal in frequency domain.

This results in the time-frequency characteristics of the SMSP interference signal, which consists of *n* straight-line segments with different intercepts in the time domain entropy with slopes of nK. Meanwhile, the width of each segment in the time domain is τ/n, and the corresponding intercept of the *i*-th segment is −(i−1)B. The time-frequency distribution of the target echo LFM signal and the SMSP interference signal is given in Figure 1. Assuming that the width of the interferer pulse is $\tau_{J}$, and the FM slope is $K_{J}$, the relationship with the transmit signal is

(20) $$\left\{\begin{array}{l} \tau_{J}=\tau /n\\ K_{J}=nK \end{array}\right.$$

Figure 1a shows that the bandwidth of the SMSP jamming signal is the same as that of the radar transmit signal, but the FM slope is different from that of the radar transmit signal. Therefore, the time-frequency characteristics are not exactly the same as those of the radar transmit signal as shown in Figure 1b. Figure 1c shows that in the frequency domain, the interference signal spectrum has completely covered the target echo signal.

**Figure 2:**
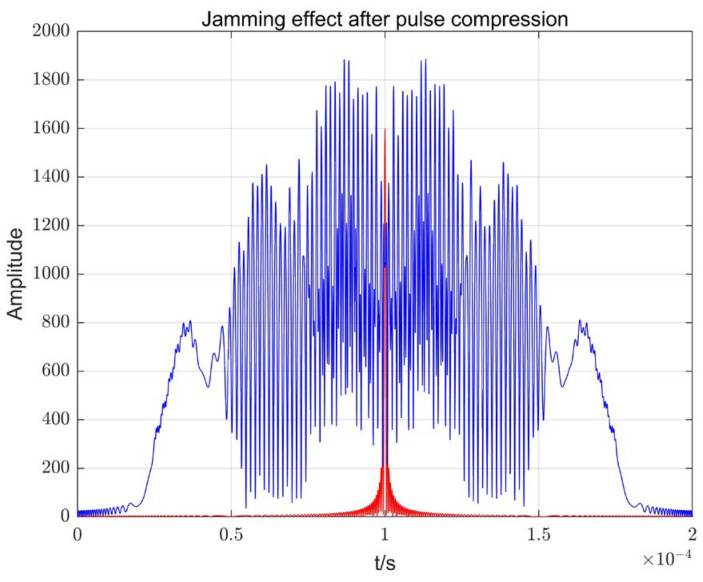
SMSP and target echo after pulse compression.

The effect of the echo signal after pulse compression processing is shown in Figure 2. As can be seen from Figure 2, the compressed LFM signal is completely drowned out by the background, and the traditional processing method is no longer applicable.

### 3.2. Maximum Entropy Estimation

Entropy is usually used to measure the uncertainty of a random variable [41]. Maximum entropy estimation (MEM) is an extension of linear prediction. In this paper, the maximum entropy algorithm is used to implement the two-dimensional angle estimation of target azimuth and elevation.

The maximum entropy estimation of the signal X received by the array is performed by filtering the spatial signal in the spatial domain. To solve the spatial domain filter W, the following optimization model is established based on the design criterion of maximizing the anti-jamming improvement factor:

(21) $$\left\{\begin{array}{l} \min W^{H}XX^{H}W\\ s.t.\;u_{0}^{T}W=1 \end{array}\right.$$

where min(·) denotes the spatial domain filter that minimizes (·), and s.t. (·) denotes the constraint. $XX^{H}$ denotes the power of the radar-received signal, $W^{H}XX^{H}W$ denotes the power of the received signal after filtering, and $u_{0}={\begin{array}{cccc} {}[1 & 0 & 0 & \begin{array}{ccc} \ldots & 0 & 0] \end{array} \end{array}}^{T}$.

When applying the Lagrange multiplier method to solve the model, set

(22) $$L(W)=\frac{1}{2}W^{H}XX^{H}W-\lambda (u_{0}^{T}W-1)$$

Taking the derivative and setting it to zero, the power vector is

(23) $$W=\mu XX^{T}u_{0}$$

Substituting (23) into the constraints of (21), with the maximum entropy algorithm proposed by Burg, the following can be obtained [42]:

(24) $$P_{MEM}=\frac{1}{{\left|a_{\theta }{\left(XX^{H}\right)}^{-1}u_{0}\right|}^{2}}$$

According to Equation (24), the target angle can be estimated as

(25) $$P(\alpha ,\beta )=arg\underset{\alpha ,\beta }{\max}\frac{1}{{\left|a_{\alpha ,\beta }{\left(XX^{H}\right)}^{-1}u_{0}\right|}^{2}}$$

Based on the maximum entropy estimation algorithm, the maximum two-dimensional information, which is the azimuth and elevation angle of the received signal, can be extracted in Equation (24). The target signal and jamming signal are both from the main beam, so at this angle, two-dimensional beamforming technology is used to suppress sidelobe jamming and enhance the mainlobe signal. Next, we will complete the extraction of the distance to the target.

### 3.3. Blind Source Separation

The blind source separation algorithm is mainly used to separate independent mixed sources. Assuming that the sources are independent of each other and the signal source is S(t), the observation matrix X(t), under the condition that the noise N(t) is considered, can be expressed as

(26) X(t)=AS(t)+N(t)

where A denotes a linear operator. The purpose of blind source separation is to find a linear operator W to reconstruct the source signal, and the reconstructed signal Y(t) is Y(t)=WX(t)+N(t).

**Figure 3:**
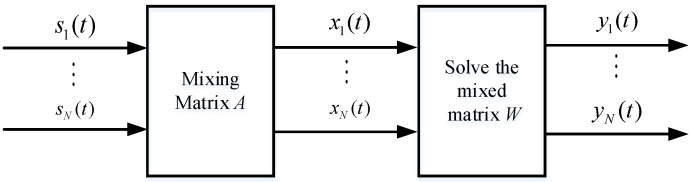
Principle of blind source separation.

The principle of blind source separation is shown in Figure 3.

In this paper, after mixing, the target echo signal and the jamming signal can be expressed as $x(t)={\left(x_{1}(t),x_{2}(t),\ldots ,x_{M}(t)\right)}^{T}\in R^{M\times T_{0}}$. The mathematical model is

(27) $$x(t)=As(t)+n(t),t=1,2,\ldots ,T_{0}$$

where $A=a_{ij}$ denotes the mixing matrix of M×M, which represents the Kronecker product of the transmit and receive steering vectors of the radar at the previously estimated angle. $n(t)={\left(n_{1}(t),n_{2}(t),\ldots ,n_{M}(t)\right)}^{T}\in R^{M\times T_{0}}$ denotes that the received signal has M noise. With the increase in the noise signal, the signal-to-noise ratio is reduced, which can seriously affect the performance of the algorithm, and the effect of blind source separation is seriously worse.

In this paper, we use the FASTICA algorithm based on the maximum signal-to-noise ratio (MSNR) to complete the blind source separation calculation. First, the error of the estimated signal Z and the source signal S is taken as the noise signal, the objective function of the maximum SNR can be obtained as

(28) $$\mathrm{SNR}=10\mathrm{lg}\frac{SS^{H}}{(S-Z){\left(S-Z\right)}^{H}}$$

The sliding average of the signal $\tilde{Z}$ is used instead of the source signal S. Then, the above equation can be expressed as

(29) $$\mathrm{SNR}=10\mathrm{lg}\frac{\tilde{Z}{\tilde{Z}}^{H}}{(\tilde{Z}-Z){\left(\tilde{Z}-Z\right)}^{H}}$$

where $\tilde{Z}(n)=\frac{1}{P}\sum_{p=0}^{P-1}Z(n-p),p=0,1,\ldots ,P-1$, P are the number of sliding averages. The resulting MSNR objective function is

(30) $$F(Z)=\mathrm{SNR}=10\mathrm{lg}\frac{\tilde{Z}{\tilde{Z}}^{H}}{(\tilde{Z}-Z){\left(\tilde{Z}-Z\right)}^{H}}$$

Assume that Z=Wy; $\tilde{Z}=W\tilde{y}$, where W is the white matrix; $\tilde{y}(n)=\frac{1}{P}\sum_{p=0}^{P-1}y(n-p)$ are the signals obtained by receiving mixed signals after sliding averaging. The whitened signal components are second-order statistically independent, where the covariance matrix of Z is given as

(31) $$R_{ZZ}=ZZ^{H}$$

Then, Equation (29) can be rewritten as

(32) $$F(W,y)=10\mathrm{lg}\frac{W\tilde{y}{\tilde{y}}^{H}W^{H}}{W(\tilde{y}-y){\left(\tilde{y}-y\right)}^{H}W^{H}}=10\mathrm{lg}\frac{WR_{yy}W^{H}}{W{\tilde{R}}_{yy}W^{H}}$$

where ${\tilde{R}}_{yy}=(\tilde{y}-y){\left(\tilde{y}-y\right)}^{H}$ denotes the signal auto correlation matrix after the sliding average. $R_{yy}=\tilde{y}{\tilde{y}}^{H}$ represents the covariance matrix of the observed signal, i.e.,

(33) $$R_{yy}=\Gamma \Lambda \Gamma^{H}=\left[\begin{array}{cc} \Gamma_{P} & \Gamma_{MN-P} \end{array}\right]\left[\begin{array}{cc} \Lambda_{P} & 0\\ 0 & \Lambda_{MN-P} \end{array}\right]\left[\begin{array}{c} \Gamma_{P}^{H}\\ \Gamma_{MN-P}^{H} \end{array}\right]$$

where

$\Lambda =\left[\begin{array}{cc} \Lambda_{P} & 0\\ 0 & \Lambda_{MN-P} \end{array}\right]\in {\mathbb{C}}^{MN\times MN}$ represents the eigenvalue matrix,
$\Gamma =\left[\begin{array}{cc} \Gamma_{P} & \Gamma_{MN-P} \end{array}\right]\in {\mathbb{C}}^{MN\times MN}$ denotes the eigenvector matrix corresponding to eigenvalues, and
$\Lambda_{P}\in {\mathbb{C}}^{P\times P}$ and $\Gamma_{P}\in {\mathbb{C}}^{MN\times P}$ are large eigenvalues and eigenvectors, respectively.
$\Lambda_{MN-P}\in {\mathbb{C}}^{\left(MN-P\right)\times \left(MN-P\right)}$ and $\Gamma_{MN-P}\in {\mathbb{C}}^{MN\times \left(MN-P\right)}$ are small eigenvalues and eigenvectors.

Because the eigenvalues in matrix $\Lambda_{p}$ are much larger than those in $\Lambda_{MN-P}$, the $R_{yy}$ can be approximately written as

(34) $$R_{yy}\approx \left[\begin{array}{cc} \Gamma_{P} & \Gamma_{MN-P} \end{array}\right]\left[\begin{array}{cc} \Lambda_{P} & 0\\ 0 & 0 \end{array}\right]\left[\begin{array}{c} \Gamma_{P}^{H}\\ \Gamma_{MN-P}^{H} \end{array}\right]=\Gamma_{P}\Lambda_{P}\Gamma_{P}^{H}$$

Substituting (34) into Equation (31), $R_{ZZ}$ can be expressed as

(35) $$R_{ZZ}=W^{H}\Gamma_{P}\Lambda_{P}\Gamma_{P}^{H}W=I_{P\times P}$$

The whitening matrix W can be obtained by solving (34)

(36) $$W={\left(\Lambda^{1/2}\Gamma^{H}\right)}^{-1}$$

Nevertheless, the components of the source signal, which are independent, cannot be restored with only the second order. In order to solve this problem, the fourth-order cumulant of the whitened signal is widely used.

**Figure 4:**
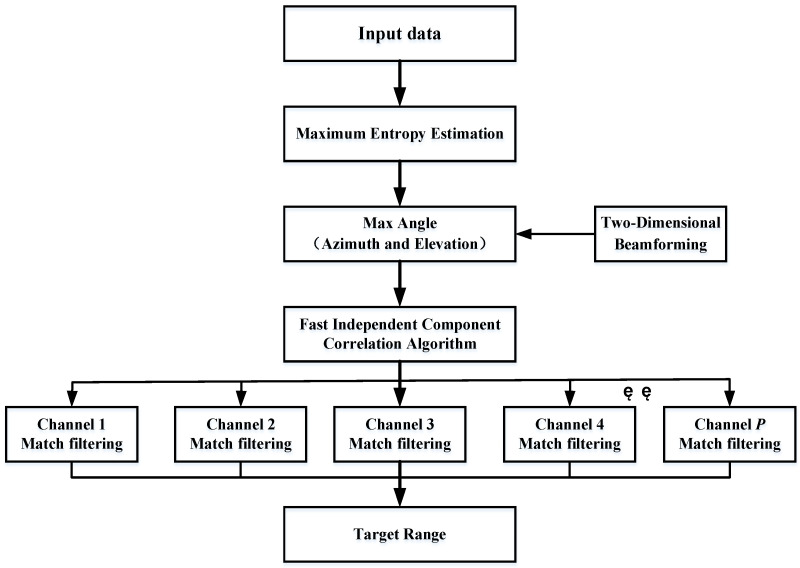
Flow chart of FDA-MIMO radar anti-SMSP.

Since the SMSP and target echo are independent of each other, the received signal matrix can be decomposed into eigenvectors to obtain *N* eigenvalues. After sorting these *N* eigenvalues, the eigenvectors corresponding to the first *L* large eigenvalues can be selected to constitute the separation matrix. The subspace corresponding to the eigenvectors of other small eigenvalues tends to be orthogonal to the mixing matrix ***A***. The *L*-th corresponds to the target echo signal, and its corresponding eigenvectors constitute *L* jamming channels. The eigenvectors corresponding to the first *L*-1 eigenvalues constitute *L* interference channels. The target channel is composed of the eigenvectors corresponding to the first *L* − 1 eigenvalues, which constitute *L* interference signals. After separating the target from the *L* channels, the pulse compression process can be completed separately, and the distance resolution unit where the target and jamming are located can be obtained, achieving the effect of suppressing the mainlobe interference. The algorithm flow of this paper is shown in Figure 4.

Step 1: The maximum entropy algorithm is used to estimate the angle of the radar echo signal, referring to Equation (23). If the target uses self-defense interference and the real target and interference signals are both in the mainlobe, only one form of angle information can be estimated. In two-dimensional beamforming, if multiple-angle information is estimated, the target and interference come from different spatial domains, and the sidelobe cancellation algorithm can be used to complete interference suppression.

Step 2: After the angle estimation is completed, the FASTICA algorithm based on the maximum signal-to-noise ratio is used. The number of channels to be separated is determined according to the number of large eigenvalues obtained from matrix decomposition. By utilizing the characteristics of the radar radiation signal and interference signal, the target and multiple SMSP signals are separated into different channels.

Step 3: Matched filtering is performed on each separated channel. The waveform of the SMSP interference signal is different from that of the LFM signal, which causes a mismatch in the interference channel. Therefore, the amplitude of the interference signal after matched filtering decreases, and the true distance of the target can be obtained.

Step 4: By utilizing the angle-distance two-dimensional coupling characteristics of the FDA-MIMO transmission vector and performing pulse compression processing, the interference signal is effectively suppressed, and the target distance information is accurately extracted.

## 4. Simulation Verification

This section analyzes and verifies the effectiveness of the proposed method through simulation. It is assumed that the radar search finds the target when receiving jamming signals from the main beam, and the airborne ECM system uses SMSP interference to suppress radar detection for radiation signals such as LFM. The effectiveness of the proposed algorithm is verified after 1000 Monte Carlo simulations. The simulation parameters are shown in Table 1.

### 4.1. Angle Estimation

**Figure 5:**
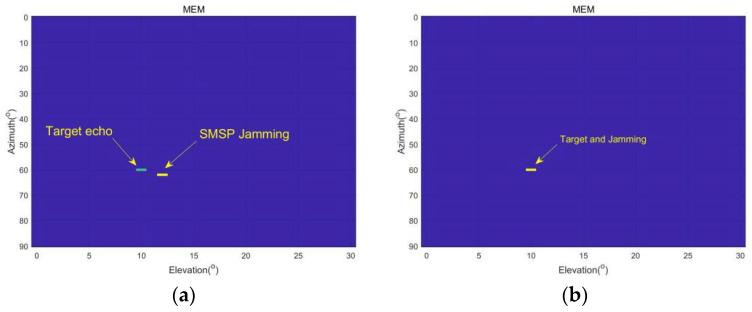
Maximum entropy estimates of two-dimensional angle information. (**a**) Jammer and target from different angles. (**b**) Jammer and target from same angles.

As shown in Figure 5a, because the amplitude of the interference signal is greater than that of the echo signal, and the target echo and SMSP signal are not in the same beam, the radar can accurately distinguish between interference and the target. As shown in Figure 5b, after the airborne electronic countermeasure system uses self-defense interference, both the interference signal and target signal come from the mainlobe, and it is impossible to accurately distinguish between the target and interference signals in this case.

### 4.2. Calculation of Similarity Coefficients

The similarity coefficient is used to measure the degree of consistency between the source signal and the separated signal [43]. In order to avoid the influence of inversion and facilitate comparison, the absolute value of the similarity system is generally calculated; that is,

(37) $$\xi_{ij}=\zeta (s_{i},y_{j})=\left|\frac{\sum_{t=1}^{M}s_{i}(t)y_{j}(t)}{\sqrt{\sum_{t=1}^{M}s_{i}^{2}(t)\sum_{j=1}^{M}y_{j}^{2}(t)}}\right|$$

where $s_{i}(t)$ is the *i*-th component of the source signal, and $y_{j}(t)$ is the corresponding *j*-th component after separation, where i,j=1,2,…,M. From this, it can be obtained that when the correlation coefficient is closer to 1, it means that the separated signal is in better agreement with the source signal, and the separation effect is higher. In this paper, it means that the separation of the target echo signal from the jamming signal is higher.

**Figure 6:**
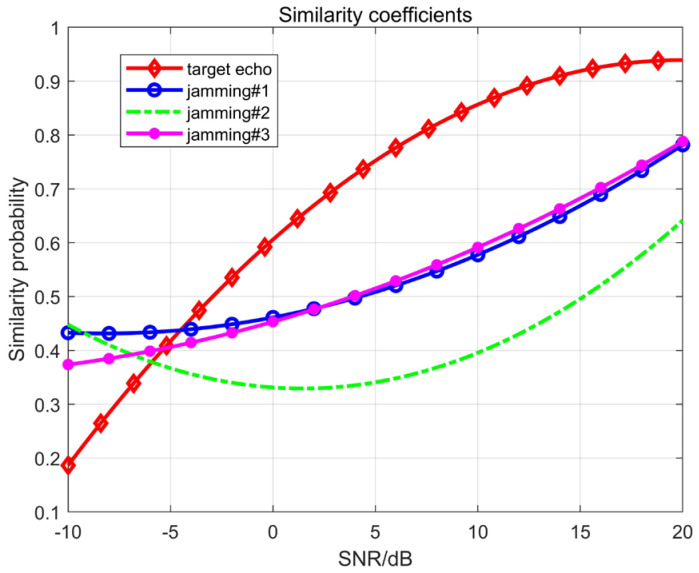
Similarity coefficients at different SNRs.

As shown in Figure 6, after blind source separation, the similarity probability of the radar target channel signal increases with the increase in the SNR. When the SNR is greater than 15 dB, the similarity approaches stability, and the probability is greater than 0.9. Due to the randomness of the interference signal being much greater than that of the echo (JSR = 20 dB), the similarity of the separated interference signal is generally lower than that of the target.

### 4.3. Comparison of before and after Blind Source Separation

**Figure 7:**
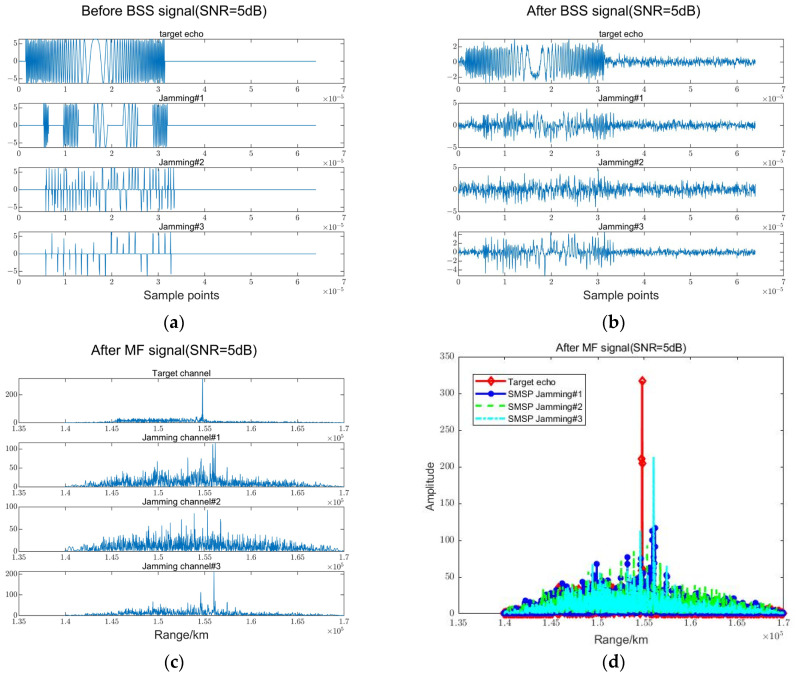
Results of match filtering after BSS (SNR = 5 dB). (**a**) The waveform diagram before BSS. (**b**) The waveform diagram after BSS. (**c**) The waveform diagram after MF (4 channel). (**d**) The amplitude after MF.

The FASTICA algorithm based on the maximum SNR is used to separate the echo signal received by the radar, but the effect of this algorithm is also affected by the power of the SNR. Figure 7 verifies the effect after blind source separation and match filtering in the case of SNR = 5 dB. Figure 7a,b show the waveform of the target echo and jamming signal before and after BSS in the four channels, respectively. Without considering the phase, the echo signal can be completely separated, but the effect of the three jamming channels is greatly affected by noise, and no significant waveform pattern can be observed. Figure 7c,d show the signal amplitude after matched filtering for each channel. It can be seen that the amplitude of the target echo signal is significantly higher than that of other signals, achieving a good interference suppression effect.

**Figure 8:**
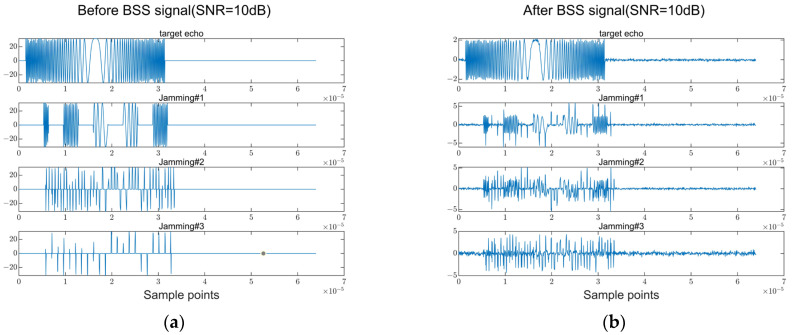
Results of match filtering after BSS (SNR = 10 dB). (**a**) The waveform diagram before BSS. (**b**) The waveform diagram after BSS.

As can be seen in Figure 8a,b, when SNR = 10 dB, after the echo signal is processed with the FASTICA algorithm, the target echo and Jamming signal can be significantly separated, and the waveforms before and after treatment are essentially consistent.

**Figure 9:**
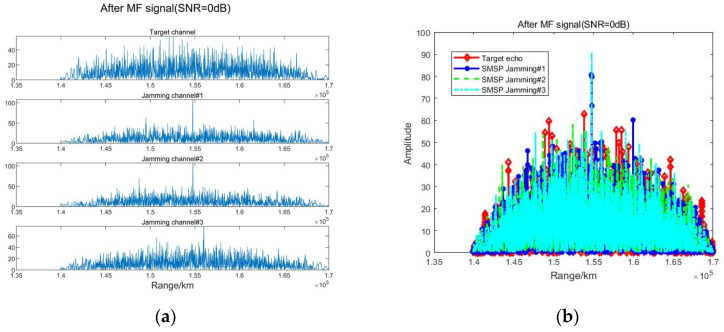
Results of match filtering after BSS (SNR = 0 dB). (**a**) The waveform diagram after MF (4 channel). (**b**) The amplitude after MF.

As shown in Figure 9, when SNR = 0 dB, the BSS algorithm is affected by noise. After match filtering for each channel, the target channel signal power is lower than the jamming channel, and the subsequent processing is prone to a false alarm with the wrong target distance.

### 4.4. Detection Probability

**Figure 10:**
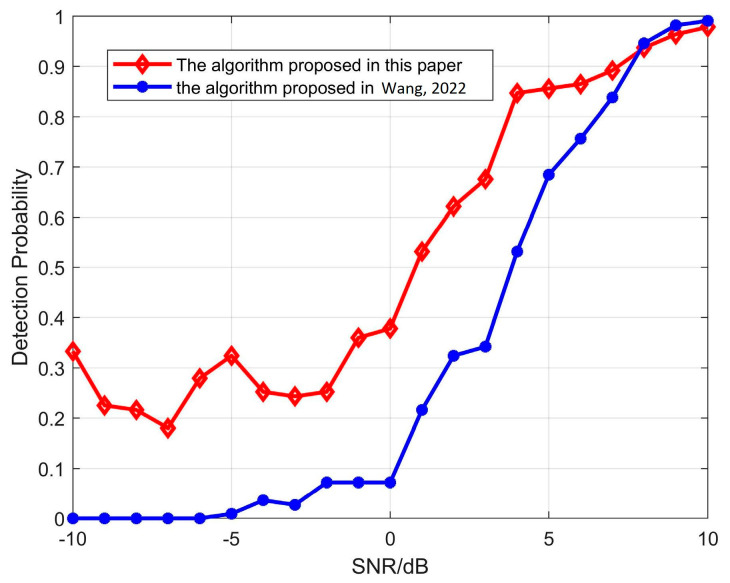
Detection probability changes with SNR [44].

Through the above analysis and simulation, it can be seen that the suppression effect of the proposed algorithm on the mainlobe jamming is affected by the signal-to-noise ratio. After 1000 Monte Carlo simulations, the detection probability of the proposed algorithm at SNRs ranging from −10 to 10 dB was statistically analyzed. As shown in Figure 10, when SNR<0dB, the detection probability is not greater than 0.4. When SNR≥0dB, the detection probability significantly increases and outperforms the maximum SNR beamforming algorithm against mainlobe interference proposed in [44]. When SNR > 9dB, the detection probability of both algorithms approaches 1, effectively suppressing mainlobe interference signals.

## 5. Conclusions

In this paper, the problem of mainlobe deceptive jamming suppression with the FDA-MIMO radar was investigated. By using two-dimensional angle and one-dimensional distance information, the transmit and receive steering vectors of the FDA-MIMO radar were developed. By analyzing the characteristics of the SMSP jamming signals and LFM signals, it is possible to separate the true and false targets corresponding to different angles or distances in the spatial domain.

In such a way, the algorithm using the maximum entropy estimation can first filter out the sidelobe jamming signal and then use the blind source separation process to effectively separate the true and false target signals; finally, taking full advantage of the difference between SMSP and LFM signals, each isolated channel is match-filtered. When the SNR reaches a certain degree, the true target position can be effectively detected, and the SMSP jamming from the mainlobe is greatly suppressed. Comparative analysis showed that the detection performance of the proposed algorithm is superior to that of the monopulse radar method.

The complexity of the proposed method needs to be further investigated due to the need to complete the three key aspects of angle estimation, ICA and MF. In the meantime, the next step will be to continue research on the suppression of multiple mainlobe jamming under low-SNR conditions, focusing on some new interference patterns and new radar systems.

## Figures and Tables

**Table 1:** Radar simulation parameters.

Parameters	Value	Parameters	Value
Operating frequency	5 GHz	Pulse re-frequency	10 kHz
Sampling frequency	5 MHz	Pulse width	32 μs
Target range	160 km	Target speed	500 m/s
Number of row elements	8	Number of array elements	8
Row element spacing	0.03 m	Array element spacing	0.03 m
Target azimuth	60°	Target elevation	10°
JSR	20 dB	Number of SMSPs	3
Monte Carlo numbers	1000		

## Data Availability

The data presented in this study are available on request from the corresponding author.
